# Efficient Hybrid Blind Watermarking in DWT-DCT-SVD with Dual Biometric Features for Images

**DOI:** 10.1155/2022/2918126

**Published:** 2022-09-08

**Authors:** Bhargavi Mokashi, Vandana S. Bhat, Jagadeesh D. Pujari, S. Roopashree, T. R. Mahesh, D. Stalin Alex

**Affiliations:** ^1^Department of Computer Science and Engineering, PES University, Bengaluru, India; ^2^Department of Information Science and Engineering, Sri Dharmasthala Manjunatheshwara College of Engineering and Technology, Dharwad, India; ^3^Department of Computer Science and Engineering, RV Institute of Technology and Management, Bengaluru, India; ^4^Department of Computer Science and Engineering, Jain (Deemed-to-be University), Bengaluru, India; ^5^Department of Computer Science and Engineering, State University of Bangladesh, Dhaka, Bangladesh

## Abstract

In the modern era of virtual computers over the notional environment of computer networks, the protection of influential documents is a major concern. To bring out this motto, digital watermarking with biometric features plays a crucial part. It utilizes advanced technology of cuffing data into digital media, i.e., text, image, video, or audio files. The strategy of cuffing an image inside another image by applying biometric features namely signature and fingerprint using watermarking techniques is the key purpose of this study. To accomplish this, a combined watermarking strategy consisting of Discrete Wavelet Transform, Discrete Cosine Transform, and Singular Value Decomposition (DWT-DCT-SVD) is projected for authentication of image that is foolproof against attacks. Here, singular values of watermark1 (fingerprint) and watermark2 (signature) are obtained by applying DWT-DCT-SVD. Affixing both the singular values of watermarks, we acquire the transformed watermark. Later, the same is applied to cover image to extract the singular values. Then we add these values to the cover image and transformed watermark to obtain a final watermarked image containing both signature and fingerprint. To upgrade the reliability, sturdiness, and originality of the image, a fusion of watermarking techniques along with dual biometric features is exhibited. The experimental results conveyed that the proposed scheme achieved an average PSNR value of about 40 dB, an average SSIM value of 0.99, and an embedded watermark resilient to various attacks in the watermarked image.

## 1. Introduction

Copyright infringement has increased as a result of the rapid blooming of cyberspace and communication technology, which has led to an exchange of digital mixed media content. The transmission of digital data across public networks like the Internet makes the protection of personal information and intellectual property rights (IPR) crucial in the modern day [1]. Digital watermarking is a means to get around this problem and prove ownership of digital assets that are being used unlawfully.

The ease of multimedia content distribution is due to the fast development of the internet, multimedia technologies, communication, and reproduction. Multimedia data is prone to issues such as illegal copying and distribution (pirating), editing, and copyright. In order to protect the data from the above-mentioned issues, digital watermarking is used.

An encrypted sort of coding called a digital watermark is added to a signal that can handle sounds, such as audio, video, or image data. Biometric systems have been using watermarking techniques to safeguard and authenticate biometric data and improve recognition accuracy in an effort to boost the trustworthiness of self-awareness systems that can be differentiated between a legitimate person and a fraudster. An encrypted sort of coding called a digital watermark is added to a signal that can handle sounds, such as audio, video, or image data. Biometric systems have been using watermarking techniques to safeguard and authenticate biometric data and improve recognition accuracy in an effort to boost the trustworthiness of self-awareness systems that can tell the difference between a legitimate person and a fraudster.

The proposed work briefs on how to authenticate images by embedding biometric information into a digital image using a new hybrid system that includes three different algorithms namely DWT-DCT-SVD. In the embedding process, the cover image undergoes a DWT transform which decomposes it into four subbands, namely, L-L, L-H, H-L, and H-H, where L-L denotes Low-Low, L-H denotes Low-High, H-L denotes High-Low, H-H denotes High-High. L-L subband undergoes DCT transform to obtain 4 × 4 blocks. The DCT transform mainly compresses the data or image. The SVD of a matrix is an orthogonal transform used for matrix diagonalization to obtain singular values of the watermark. Subsequently, the SVD factors of each block are modified to create the watermarked image, extracted, and then inserted into the cover image. In the process of extraction, the watermarked image is acquired and a reverse stratagem is utilized to obtain the watermark, which is the biometric data.

**Figure 1:**
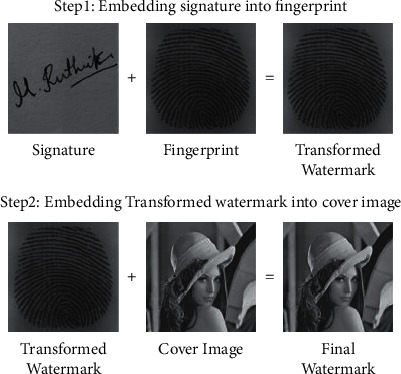
Embedding process of the watermark image.

Biometrics refers to the automatic identification of people based on their physiological and behavioral features; two authentications based on behavioral and physiological characteristics for attaching the watermark to the cover image are applied. Measurements taken from the human body are used in physiological biometrics, such as fingerprints, iris, face, retina. The dynamic measurements used in behavioral biometrics such as signatures, voice, and keystrokes, are based on human actions. The proposed hybrid watermarking system is cooperative integration of signature and fingerprint watermarks to cover image to assure the integrity, authenticity, and confidentiality of the digital documents. The embedding procedure consists of two steps in the projected method. First, the embedding of the signature in the fingerprint is carried out to create the transformed watermark, as shown in Figure 1. The final watermark is created by embedding the cover image in this phase.

**Figure 2:**
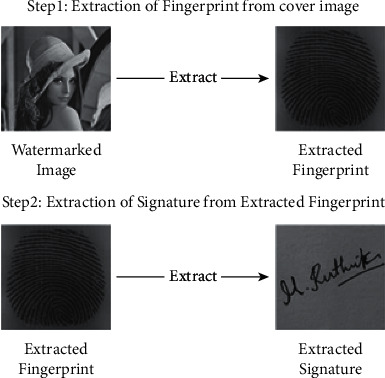
Extraction process of the watermark image.

The extraction procedure is split into two steps. Step 1: extract the fingerprint from the watermark that results in an extracted fingerprint. Step 2: the signature is further extracted from the extracted fingerprint image, as shown in Figure 2.

### 1.1. Hybrid DWT-DCT-SVD

The proposed scheme consists of DWT, DCT, and SVD for image authentication that is robust against attacks. In the process of watermarking, two major steps are carried out viz., embedding and extraction. In this, the combinations of DWT, DCT, and SVD along with their inverses are applied. This hybrid technique is suitable for different image processing attacks by achieving the properties of watermarks, i.e integrity, authenticity, and confidentiality of digitized image documents. The performance metrics used in this research are Peak Signal to Noise Ratio (PSNR), Structural Similarity Index (SSIM), and Normalized Correlation (NC). This proposed methodology is deployed on dual watermarking where the embedding process consists of DWT, DCT, and SVD which provide image authentication and is robust against attacks.

The embedding process consists of DWT, DCT, and SVD watermarking techniques. To cover the image, one level of DWT is applied. Hence applied SVD to the L-L sub-band. Besides, the application of DWT to the biometric and then DCT followed by the SVD technique is carried out. Parallelly, SVD is applied to the signature. Application of SVD to the images results in three matrices namely U S and V. Considered the singular valued S matrix as it contains the diagonal properties of the image. Further, added the singular values of the biometric and alpha times of the signature. To recreate the L-L sub-hand of biometric the inverse of the SVD is applied. Later, we applied inverse DCT as we applied DCT in the earlier steps. Now we have applied inverse DWT to create an image with a modified L-L subband. This gives a results in the transformed watermark. Now apply the application of SVD to it in order to get a singular valued matrix. Next, to cover the image, singular values are added off and beta times singular matrix of the transformed watermark. Now apply the inverse SVD to recreate the cover image with manipulated singular values. Then followed by applying DCT and then DWT to create an image with a modified L-L subband. This gives a final watermarked image; this contains the signature and biometric embedded on the cover image, and this completes the embedded process. The extraction process for the transformed watermark (biometric) is done by applying DWT on the final watermark to obtain four subbands. Next, apply DCT to the L-L sub-band followed by SVD to obtain singular values of final watermarked image. Later, DWT is followed by DCT and then SVD to obtain signature images from the transformed image. This completes the extraction process.

### 1.2. DCT

When digital photos are uncompressed, they require a massive quantity of storage space. For such uncompressed data to be transmitted across the network, large transmission bandwidth is required. The most common image compression method is the Discrete Cosine Transform (DCT) [1]. The JPEG picture compression method makes use of DCT. The two-dimensional DCT is calculated for each block of the 8 × 8 or 16 × 16 divided input image. Following that, the DCT coefficients are quantized, encoded, and transferred.

The DCT can store the image with only fewer coefficients, and is used in lossy image compression to reduce the redundancy between neighboring pixels. The DCT formula with a 2D matrix is shown in equation (1).

(1) $$\begin{array}{c} D\left.\left(i,j\right)=\frac{1}{\sqrt{2N}}C\left(i\right)C\left(j\right)\overset{N-1}{\underset{x=0}{\sum }}\overset{N-1}{\underset{y=0}{\sum }}p\left(x,y\right)\cos\;\left[\frac{\left(2x+1\right)i\pi }{2N}\right]\;\cos\;\left[\frac{\left(2y+1\right)j\pi }{2N}\right.\right], \end{array}$$

where the *x*, *y*^th^ elements of the image element are represented by the matrix *p* as *p*(*x*, *y*). The block's size, *N*, is used for the DCT. The pixel values of the native matrix of the image equation determine the value of one entry (*i*, *j*^th^) of the modified image. For the standard JPEG 8 × 8 blocks, *N* = 8 and (*x*, *y*) is in the stretch of 0 to 7.

The DCT divides pictures into components with various frequencies. Because fewer significant frequencies are dropped during quantization in the compression portion, the term lossy is in use. Later, during the decompression phase, the image is retrieved using the remaining most crucial frequencies. As a result, some distortion is included in the reconstructed images; however, the levels of distortion can be altered during the compression stage. JPEG is used for both color and black and white photographs; however, the article focuses on the latter.

### 1.3. DWT

**Figure 3:**
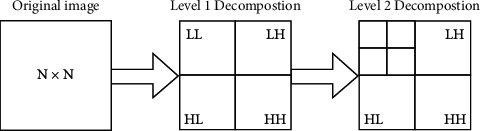
Subbands resulting after 2-level decomposition.

The suggested methodology incorporates the Discrete Wavelet Transform (DWT) [2] approach to withstand the attacks with a robust model. Low-Low, Low-High, High-Low, and High-High, i.e L-L, L-H, H-L, and H-H are four subbands created by DWT (HH). The original image will be recreated using the above four subbands. The image can theoretically be processed via the filter bank as shown in Figure 3 to produce various subband frequency images.

**Figure 4:**
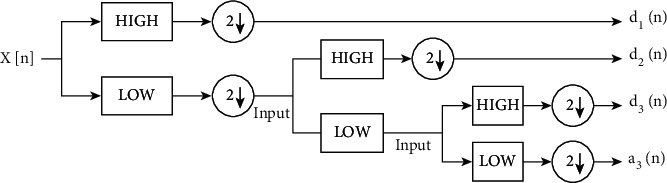
Block diagram of the 2-level DWT scheme.

As illustrated in Figure 4, the L-L subband defines low-pass filtering for each row and column, resulting in a low-resolution approximation of the original image. Similarly, the L-H subband was created by applying low-pass filtering to each row and high-pass filtering to each column. The L-H subband is influenced by high-frequency features along the column direction. The H-L subband is the result of high-pass and low-pass filtering on each row and column. The H-L subband is influenced by high-frequency features along the row direction. The H-H subband is created by applying high-pass filtering to each row and column. The H-H subband is influenced by high-frequency features in the diagonal direction [3].

DWT-Based Feature Extraction: using multilevel decomposition of previously processed pictures, DWT effectively extracts discriminant characteristics that are impervious to arbitrary environmental fluctuations. The discrete interval wavelets are sampled for the wavelet transform known as the DWT. DWT provides information about the frequency and spatial domains of a picture simultaneously. An image can be studied using the DWT operation, which combines the analysis filter bank and decimation process. A 2D transform is created from two distinct 1D transformations. In 1D DWT, the approximation coefficients hold the low-frequency information, whereas the detail coefficients hold the high-frequency information. The input image is divided into four separate subbands by the application of 2D DWT: low-frequency components in the horizontal and vertical directions (cA), low-frequency components in the horizontal and high-frequency components in the vertical directions (cV), high-frequency components in the horizontal and low-frequency components in the vertical directions (cH), and high-frequency components in the horizontal and vertical directions (cD). You can alternatively write cA, cV, cH, and cD as L-L, L-H, H-L, and H-H, respectively.

### 1.4. SVD

Singular value decomposition (SVD) [1, 4] is a method for approximating data matrix decomposition into an optimal approximation of the signal and noise components. This is one of the most essential aspects of the SVD decomposition in noise filtering, compression, and forensics, and it can also be viewed as a properly identifiable noise addition.

SVD refactors into three matrices for the given digital image. To refactor the image singular values are used and at the end of this process storage space required by the image is reduced as the image is represented with a smaller set of values. The SVD of *M* × *N* matrix *A* is given by the following equation (2).

(2) $$\begin{array}{c} SVD=UV^{T}W, \end{array}$$

where *U*: *M* × *N* matrix of the orthonormal eigenvectors of *AA*^*T*^. 𝑉^𝑇^: Transpose of the *n* × *n* matrix containing the orthonormal eigenvectors of *A*^{*T*}*A*. *W*: *N* × *N* diagonal matrix of the singular values which are the square roots of the eigenvalues of 𝐴^𝑇^𝐴.

The system can be divided into a number of linearly independent components, each of which contributes its own amount of energy, using the most efficient and stable technique known as SVD.

The orthogonal matrix columns *U* are referred to as the left singular vectors, whereas the orthogonal matrix columns V are referred to as the right singular vectors. The diagonal members are reflecting the singular values of the matrix.

**Figure 5:**
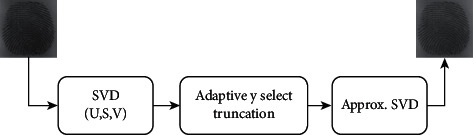
Block diagram of SVD.

The maximum energy packing of the SVD, the ability to solve the least squares issue, the ability to compute the pseudoinverse of a matrix, and multivariate analysis are all significant benefits for images [1, 5]. A crucial characteristic of SVD is its relationship to a matrix's rank and its capacity to approximate matrices of a particular rank. Digital images can frequently be characterized by the sum of a relatively limited number of Eigen images since they are frequently represented by low-rank matrices. Images are compressed in compression, and SVD with the highest energy packing property is typically used. As previously established, SVD divides a matrix into orthogonal parts so that the best sub-rank approximations can be made [6, 7]. Truncated SVD transformation with rank *r* offers significant storage savings over storing the entire matrix with acceptable quality. The block diagram for the SVD-based compression is shown in Figure 5.

First, illumination data can be found in the singular value matrix produced by SVD. As a result, altering the single values will directly impact how the image is illuminated. As a result, the image's other details won't be altered. Second, by using the L-L subband illumination enhancement, the edge information in other subbands will be protected (i.e., L-H, H-L, and H-H).

## 2. Related Work

The study [1] the research that is being offered displays an adaptive scaling factor based on particular DWT-DCT coefficients of its image material. The role of particular DWT-DCT coefficients relative to the average value of DWT-DCT coefficients was used to construct the adaptive scaling factor. Using a suggested set of guidelines that consider the adaptive scaling factor, the watermark image was integrated. The results of the experiments showed that the suggested method produced a high PSNR value of 47 dB, an SSIM value of around 0.987, and an implanted watermark resistance to many attacks in the watermarked image.

Before the integration procedure in the article [5], a discrete wavelet transform is applied to the image, and then the ZigZag scanning method is used to topologically reorganize the coefficients of the L-L subbands. The watermark bits are then integrated using the resulting coefficients. The integrity of the watermark may be easily confirmed thanks to an embedded hash of the electronic patient record. The experimental results show that the approach has high invisibility (with a PSNR above 70 dB) and very good robustness against a wide range of geometric and destructive attacks. The invisibility and robustness of the approach have been tested.

Since many of the currently used hybrid SVD-based picture watermarking systems is insecure, the study [4] primarily focuses on the analysis of the state-of-the-art in this area. Additionally, there aren't many in-depth reviews in this field. In order to draw attention to numerous security risks, unresolved challenges, and research gaps, they conducted efficiency comparisons. Based on the results, this study gives researchers and practitioners important information they can use to improve the field of picture watermarking. It also gives suggestions for how to make more reliable schemes in the future.

The work [8] achieved a superior imperceptibility of 57.6303 dB, and demonstrates that watermarking may be included in a host image using various transform operations, including discrete cosine transform (DCT), discrete wavelet transforms (DWT), and singular value decomposition (SVD). But not every design criterion is met at once by a single transformation. In order to close this gap, they developed a hybrid blind digital image watermarking technique using DCT, DWT, and SVD. This method was more robust than existing state-of-the-art techniques against filter, salt-and-pepper noise (SPN), and rotation attacks. The WNC value for a median filter with various window sizes is 1, which is higher than the current approaches.

Three well-known transforms—the discrete wavelet transform (DWT), discrete cosine transform (DCT), and singular value decomposition—are combined in the system in [6] (SVD). By reaching greater values of imperceptibility in the form of PSNR with a value of 44.0567 decibels (dB) and SSIM with a value of 0.9800, experimental results show that the suggested technique exceeds the strategies already published in the literature. With a maximum NCC value of 1.000 and a minimum BER value of 0.000, it simultaneously achieves exceptional robustness ratings. The DWT-SVD performance suggested in the study [9] was verified throughout the training phase, and the suggested system's high invisibility and resilience against different forms of attacks on watermarked photos were also demonstrated. When the suggested system's findings were contrasted with those of other systems, it became clear that DWT-SVD performed better against pixel-value alteration attacks.

The suggested work in [10] illustrates a robust watermarking technique for grayscale photos using lifting wavelet transform and singular value decomposition as the basis for multiobjective artificial bee colony optimization. Here, the actual image is changed to four subbands using three levels of lifting wavelet transform, and then the watermark image's singular value is merged with the original image's unique value for the L-H subband. In order to achieve the highest possible robustness without compromising watermark clarity, multiple scaling factors are used in the embedding operation on behalf of the single scaling element. The results of the experiments show that the invisibility is very good and that it is resistant to a wide range of attacks that use image processing. A non-blind watermarking (NBW) schemes malfunction for watermarking stratagem thereby giving out to impart perpetually imperceptibility, depriving of robustness and competence for embedding. So, to tame this drawback, an algorithm for blind watermarking (BW) was proposed [11] to cover the glitches of NBW.

To impart safeguarding of copyright that has crucial demand for color images, an image-watermarking scheme deployed on sequence-based MRT (SMRT) was tendered for color images [12] where the principle goal was to detect preferable color space among the habitually pre-owned color spaces. A cascaded neural network approach deployed on two different neural network models was projected [13] by using an optimized feature-based digital watermarking algorithm. Here, the cascading of the neural network spawns the potent pattern for embedding. In the study [14], researchers tendered a strategy using watermarking technique of Fourier transform for color images where image will be declined into two variants where the image is segmented into *R*, *G* and *B*, sections where DFT is performed and these coefficients so obtained will use medium frequency band to encapsulate WM.

In [15], which comprises of discrete wave transformation technique combined with Hessenberg decomposition (HD) and singular value decomposition (SVD) using scaling factor, watermark is embedded into the cover image. In [16], a watermarking algorithm of the color image is projected, where it explores the combination of DWT-DCT-SVD. Here the host image which is in RGB space is converted to YUV color space. Then a layer of DWT is put into the luminance component Y, followed by DCT and SVD to each block. The results are good enough to embrace the attacks and imperceptibility property of watermark. In [2, 3, 7, 17], some basic comparison of watermarking with steganography and a summary of different methods of image steganography is carried out. An effective DWT–SVD is deployed with self-adaptive differential evolution (SDE) algorithm for image watermarking scheme, SDE adjusts the mutation factor *F* and the crossover rate *Cr* dynamically in order to balance an individual's exploration and exploitation capability for different evolving phases to achieve invisibility [18–20]. In [21–24], comparative analysis of image compression is done by three transform methods, which are Discrete Cosine Transform (DCT), Discrete Wavelet Transform (DWT) and Hybrid (DCT + DWT) Transform, thereby achieving better invisibility property and good PSNR ratio.

## 3. Proposed Methodology

**Figure 6:**
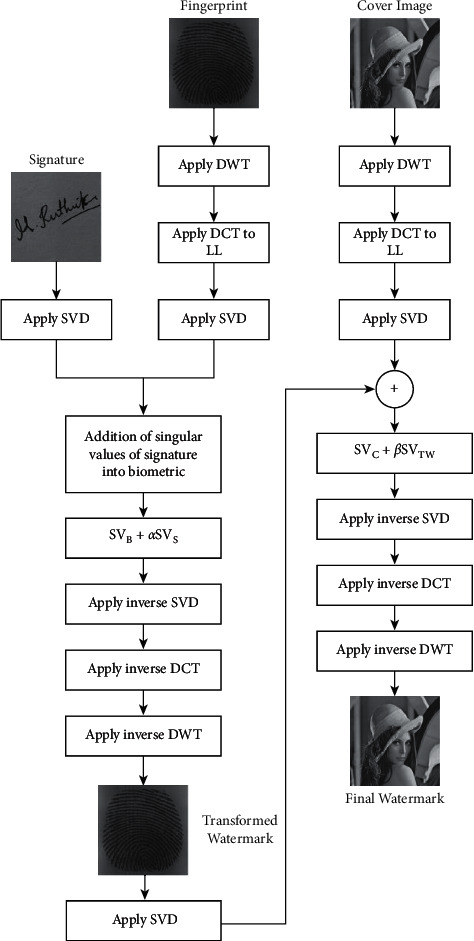
Watermark embedding using DWT-DCT-SVD and its inverse techniques.

This proposed methodology is deployed on dual watermarking where the embedding process consists of DWT, DCT, and SVD which provide image authentication and is robust against attacks.  Figure 6 depicts the embedding process that consists of DWT, DCT, and SVD watermarking techniques. The two watermarks used in the proposed methodology are biometrics and signature. These images are converted in grayscale because the SVD can only be applied to two-dimensional images whereas the color images are of three dimensions. Since the property of DWT after one level decomposition, the host image should be larger than the watermark. For the first embedding process, biometrics is the host image and the signature is the watermark. The biometric should be larger than the signature. Here, to the cover image one level of DWT is applied. Then the image is divided into four subbands, namely, L-L, L-H, H-L, and H-H. The major details and properties of the image are stored in the L-L subband. So, we contemplate embedding the biometric into the L-L subband. So, we have applied SVD to the L-L subband. Besides we have applied DWT to the biometric and then DCT and followed by SVD. Parallelly, we applied SVD to the signature, by applying SVD to the images we obtain three matrices namely U S and V.

The proposed methodology is divided into two steps:

A. Watermark Embedding Algorithm
B. Watermark Extraction Algorithm

### 3.1. Watermark Embedding Algorithm

The Embedding algorithm can be split into two phases:

1. Embedding process of signature into biometric:
    Step 1: Apply SVD to the signature to obtain the singular values SVS.
    Step 2: Apply DWT level-1 to the biometric to obtain 4-subbands.
    Step 3: Apply DCT to L-L subband in order to remove redundancy.
    Step 4: Apply SVD to the biometric to obtain singular values SVB.
    Step 5: Change the singular values of biometric SVB by adding the singular values of signature SVS.
    Step 6: The Transformed watermark TW is obtained by applying inverse SVD, DCT and DWT.
2. Embedding process of Transformed watermark into Cover image:

  Step 1: Apply DWT to cover image to obtain 4-subbands.
  Step 2: Apply DCT to L-L subband in order to remove redundancy.
  Step 3: Apply SVD to obtain the singular values of cover image SVC.
  Step 4: Manipulate the singular values of cover image SVC by adding the singular values of transformed image SVTW.
  Step 5: Obtain the final watermarked image by applying the inverse of SVD, DCT, and DWT techniques on the modified matrix

### 3.2. Extraction Process

**Figure 7:**
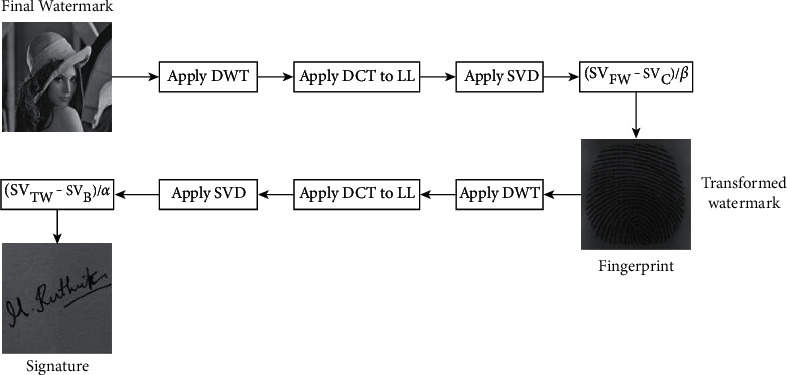
Watermark extraction using DWT-DCT-SVD and its inverse techniques.

Figure 7 depicts the extraction process, which is the extraction of watermarks, i.e., biometric and signature from the cover image. The extraction is carried out as follows:

1. Extraction of Transformed watermark (i.e, biometric):
    Step 1: Apply DWT on the final watermark to obtain four subbands.
    Step 2: Apply DCT to L-L subband in order to remove redundancy.
    Step 3: Apply SVD to obtain the singular values of the final watermarked image SVFW.
    Step 4: To obtain the transformed watermark image, subtract the singular values of final watermarked image SVFW from the cover image singular values SVC. and divide the whole with the beta parameter.
2. Extraction of signature watermark from transformed watermark (biometric):

  Step 1: Apply DWT on transformed watermark to obtain four subbands.
  Step 2: Apply DCT to L-L subband in order to remove redundancy.
  Step 3: Apply SVD to obtain the singular values of the transformed watermark.
  Step 4: To obtain a signature, subtract the singular values of transformed watermark SVTM from the biometric singular values SVB. and divide the whole with the alpha parameter.

## 4. Experimental Results

The outcome of the projected technique discloses a hybrid combination of DWT-DCT-SVD that gives the best NC values along with good PSNR and SSIM. By applying DWT alone, the host image doesn't withstand a few attacks. So, by introducing DCT, it has the ability to pack most of the information in the fewest coefficients thereby reducing the redundancy between the neighboring pixels. By using SVD, it makes it easier to hide the image. This combination works for all sorts of attacks and also gives better results.

**Figure 8:**
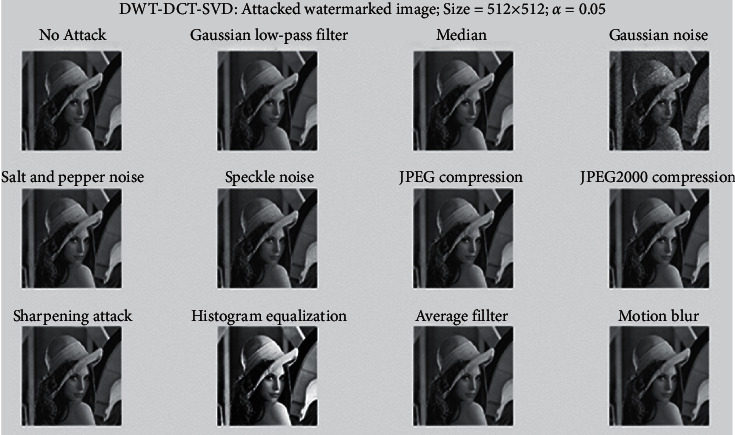
Attacked final watermark image.

**Figure 9:**
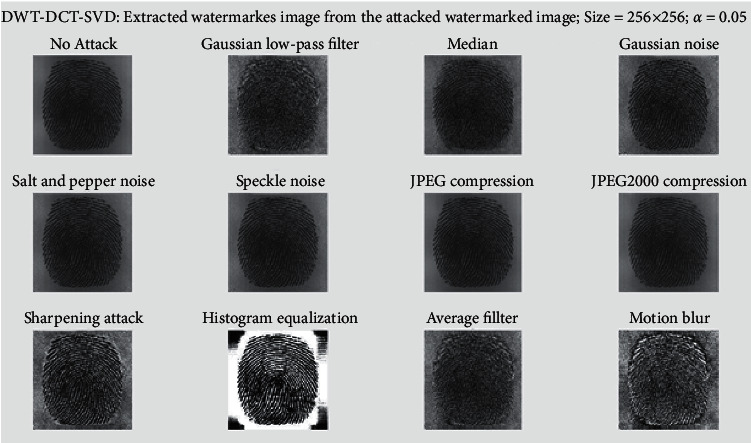
Extracted biometric image from attacked final watermark image.

In Figure 8, a watermarked image of size 512 × 512 has been subjected to various watermarking attacks, including Gaussian low-pass filter, Median, Salt and Pepper noise, Speckle noise, JPEG compression, Sharpening attack, Histogram equalization, Average filter, Gaussian noise, JPEG2000 compression, and Motion blur. It was robust against all of these attacks.  Figure 9 shows an extracted fingerprint of size 256 × 256. When the cover image is subjected to various watermarking attacks such as Gaussian low-pass filter, Median, Salt and Pepper noise, Speckle noise, JPEG compression, Sharpening attack, Histogram equalization, Average filter, Gaussian noise, JPEG2000 compression, and Motion blur. It is resistant to all of these attacks.

**Figure 10:**
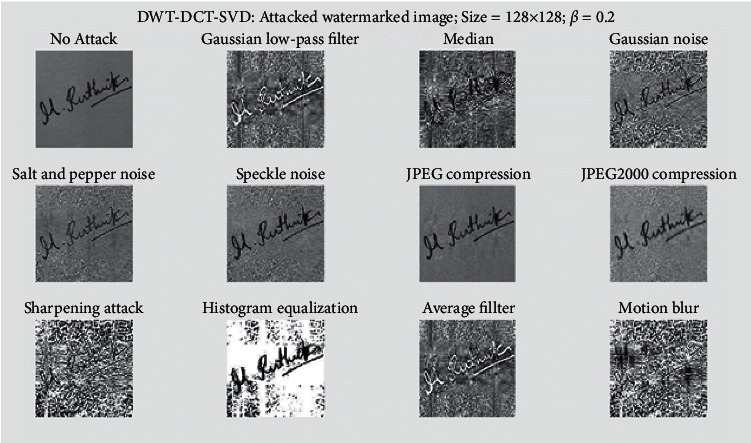
Extracted signature from attacked biometric image.

**Figure 11:**
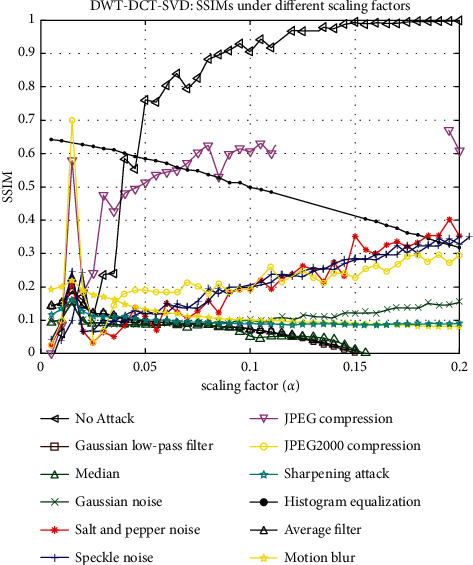
SSIMs under different scaling factors.

In Figure 10, the cover image is subjected to various watermarking attacks, such as the Gaussian low-pass filter, Median, Salt and Pepper noise, Speckle noise, JPEG compression, sharpening attack, Histogram equalization, Average filter, Gaussian noise, JPEG2000 compression, and Motion blur, an extracted signature of size 128 × 128 is displayed. It resisted all of these attacks. The graph of SSIM versus scaling factor (*α*) is shown in Figure 11. This graph depicts the behavior of SSIM values for various *α* values. Each line on the graph represents a different attack, such as a Gaussian low-pass filter, a Median, Salt and Pepper noise, Speckle noise, JPEG compression, sharpening attack, histogram equalization, an average filter, Gaussian noise, JPEG2000 compression, and motion blur.

**Figure 12:**
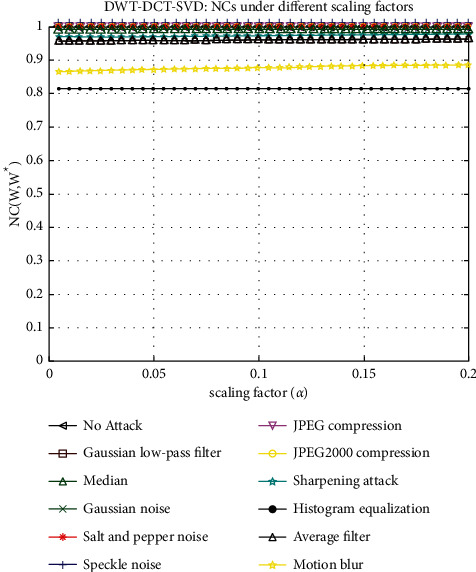
NCs under different scaling factors.

**Figure 13:**
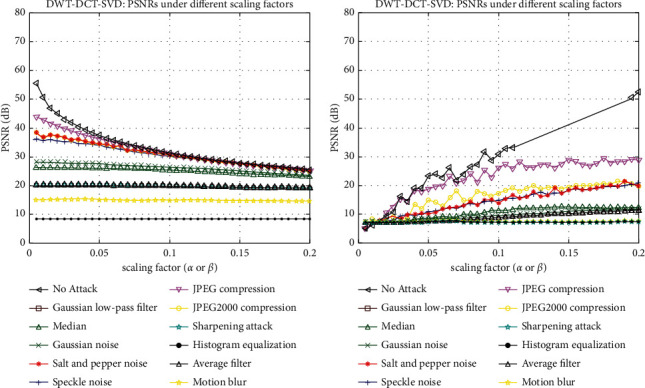
(a) PSNRs under different scaling factors. (b) PSNRs under different scaling factors.

**Figure 14:**
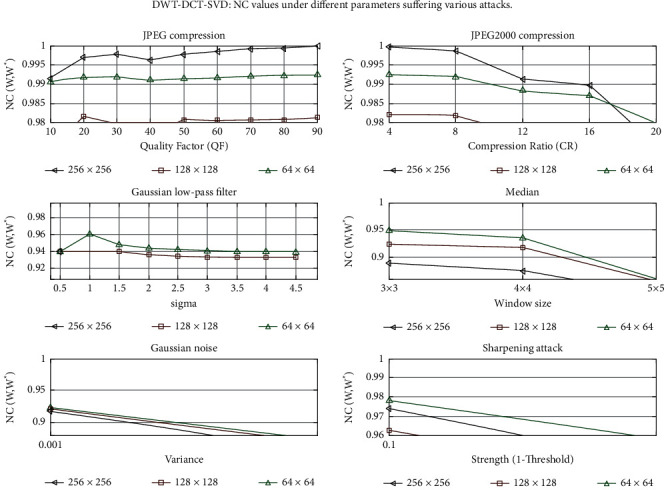
NC values under different parameters suffering various attacks.

The graph of NC versus scaling factor (*α*) is shown in Figure 12. This graph depicts the behavior of NC values for various *α* values. Each line on the graph represents a different attack, such as a Gaussian low-pass filter, a median, salt and pepper noise, speckle noise, JPEG compression, sharpening attack, histogram equalization, an average filter, Gaussian noise, JPEG2000 compression, and motion blur. Figures 13(a) and 13(b) show a graph of PSNR versus different scaling factors (*α* or *β*)). This graph shows the behavior of PSNR values for different *α* or *β* values. A Gaussian low-pass filter, a Median, Salt and Pepper noise, Speckle noise, JPEG compression, sharpening attack, Histogram equalization, an Average filter, Gaussian noise, JPEG2000 compression, and Motion blur are all represented by lines on the graph.  Figure 14 depicts graphs of NC values under various parameters subjected to various attacks. Each line in the graphs represents a different image size, such as 512 × 512, 256 × 256, and 128 × 128. The *X*-axis parameters are a quality factor, compression ratio, sigma, window size, variance, and strength (1- Threshold). The graph varies depending on the type of attack used.

Table 1 shows Normalized Correlation (NC) values for biometric (NCB) and signature (NCS) under different types of attacks. The achieved results show better NC values for all the test cases even after the extraction of watermarks (biometric and signature).

Table 2 details the invisibility (imperceptibility) property of the watermark of the proposed watermarking scheme for different types of images. It clearly shows that the proposed algorithm for all seven images showcases an average PSNR value of 39.5 and an average SSIM value of 0.99.

Table 3 depicts Peak Signal to Noise Ratio (PSNR) values for biometric (PSNRB) and signature (PSNRS) under different types of attacks. In the above-mentioned test cases, the results acquired are with good PSNR values even after the extraction of watermarks (biometric and signature).

Table 4 depicts Structural Similarity Index Metrics (SSIM) values for biometric (SSIMB) and signature (SSIMS) under different types of attacks. For all the above-mentioned test cases, the results achieved are with good SSIM values even after the extraction of watermarks viz, biometric, and signature.

Table 5 shows the NC values of various watermarked images (host image) where the two watermarks (biometric and signature) are embedded. The NC values are good enough to achieve the property of imperceptibility of both the watermarks. The table details that the proposed scheme shows comparatively good results on Lena image for crop, salt & pepper, and speckle attacks. The proposed scheme shows results on other attacks such as rotation and scaling attacks. For peppers image, the proposed scheme shows similar results to the related work [1]. It can be depicted from Table 5 that the proposed methodology (DWT-DCT-SVD) shows comparatively good results for all the 15 different types of attacks on Lena and Pepper images.

## 5. Conclusion

This study extends a watermarking stratagem deployed on both transform (DCT-DWT) and spatial (SVD) domain methods. Watermarked image implementation has good PSNR, NC, and SSIM due to DCT's energy compaction property and DWT has a better compression ratio. The results show that the proposed method besides being protective against attacks, and deployed method improves performance without sacrificing image information. The robustness of the projected watermarking strategy was assessed by performing attacks such as added noise, filtering attacks, geometrical attacks, and compression attacks. The deployed method was validated with regard to the imperceptibility of the watermarked image. The deployed method exhibits the experimental results which achieved an average PSNR of 40 dB value, an NC value of 0.9, and an SSIM value of approximately 0.99. In the future, more enhanced embedding techniques may be deployed to improve the standard of watermarked images meanwhile taking the flaws into account. In the future, this method can be improved by combining it with other watermarking techniques that are more conscientious and resistant to attack. The proposed method can embed a watermark into standard digital media such as audio, text, zip archives, and video, as well as holograms and 3D vector objects. This work can be expanded to conceal user data and personal documents.

## Figures and Tables

**Table 1:** Normalized correlation values.

#	Attacks	NCB	NCS
1	Crop	0.4345	0.9922
2	Salt & pepper	0.7586	0.7862
3	Gaussian	0.7472	0.7002
4	Speckle	0.4865	0.8047
5	Rotation	0.5005	0.8827
6	Scale 2X	0.9590	0.9340
7	Scale 0.5X	0.6307	0.7165
8	Median	0.9650	0.9751
10	Sharpening	0.5507	0.6234
11	Motion blur	0.6217	0.8421
12	Average filter	0.62	0.8090
13	Histogram equalization	0.6012	0.6104
14	JPEG	0.857	0.846
15	JPEG2000	0.846	0.8562

**Table 2:** Invisibility of proposed watermarking scheme for seven images.

#	Host image title	Image	PSNR	SSIM
1	Lena	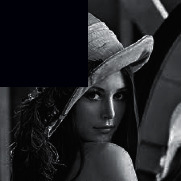	39.4843	0.9964

2	Women_Dark hair	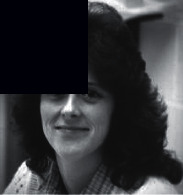	39.5767	0.9934

3	Mandrill	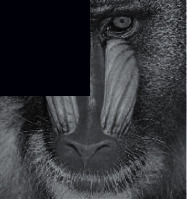	39.4837	0.9967

4	Peppers	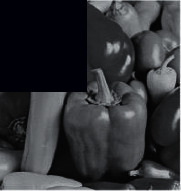	39.4820	0.9943

5	Livingroom	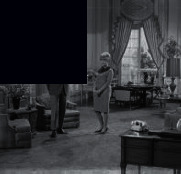	39.4835	0.9961
6	Pirate	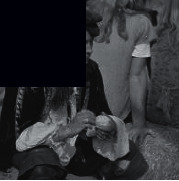	39.4827	0.9958

7	Women blonde	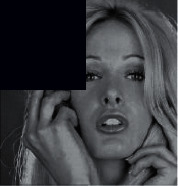	39.4861	0.9969

**Table 3:** PSNR values for biometric (PSNRB) and signature (PSNRS).

#	Attacks	PSNRB	PSNRS
1	Crop	4.1422	23.5847
2	Salt & pepper	5.2669	6.9213
3	Gaussian	6.8711	7.3798
4	Speckle	5.4516	11.4783
5	Rotation	9.0110	7.2282
6	Scale 2X	24.0391	17.1220
7	Scale 0.5X	5.5592	7.5178
8	Median	6.6279	7.2673
9	Sharpening	4.7371	9.6499
10	Motion blur	4.3715	7.5336
11	Average filter	4.3984	7.8970
12	Histogram equalization	5.4978	10.2860
13	JPEG	5.4542	9.5644
14	JPEG2000	6.4532	10.8961

**Table 4:** Structural similarity index metrics (SSIM).

#	Attacks	SSIMB	SSIMS
1	Crop	0.87634	0.90724
2	Salt & pepper	0.99536	0.76606
3	Gaussian	0.46688	0.55715
4	Speckle	0.78373	0.84695
5	Rotation	0.78654	0.49736
6	Scale 2X	0.98742	0.97214
7	Scale 0.5X	0.99146	0.98632
8	Median	0.97755	0.76953
9	Sharpening	0.72219	0.68434
10	Motion blur	0.14037	0.049129
11	Average filter	0.12215	-ve
12	JPEG	0.9519	0.94418
13	JPEG2000	0.80577	0.84715

**Table 5:** Comparison of the robustness of the proposed work with related work.

#	Image attacks	Lena		Peppers	
		Ernawan et. al., [1]	Proposed scheme	Ernawan et. al., [1]	Proposed scheme
		NC	NC	NC	NC
1	No attack	1.000	1.000	1.000	1.000
2	Crop	0.973	**0.9745**	—	0.9821
3	Salt & pepper	0.905	**1.000**	1.000	1.000
4	Gaussian	1.000	0.9988	0.9988	0.9941
5	Speckle	0.818	**0.9999**	0.9999	0.9999
6	Rotation (10°)	—	**0.75642**	—	**0.7714**
7	Scale 2X	—	**0.98712**	—	**0.9901**
8	Scale 0.5X	—	**0.99835**	—	**0.9942**
9	Median	0.999	0.9999	0.9999	0.9999
10	Sharpening	1.000	0.999	0.999	0.999
11	Motion blur	—	0.997	0.997	0.997
12	Average filter	1.000	0.999	0.999	0.999
13	Histogram equalization	1.000	0.985	0.985	0.923
14	JPEG	1.000	0.999	0.999	0.999
15	JPEG2000	0.994	0.999	0.999	0.999

## Data Availability

The dataset used for the findings can be obtained from the corresponding author upon reasonable request.
